# Genetic and genomic characterization of vulva size traits in Yorkshire and Landrace gilts

**DOI:** 10.1186/s12863-020-0834-9

**Published:** 2020-03-12

**Authors:** Flor-Anita Corredor, Leticia P. Sanglard, Richard J. Leach, Jason W. Ross, Aileen F. Keating, Nick V. L. Serão

**Affiliations:** 1 Department of Animal Science, Iowa State University, IA50010, Ames, USA; 2 Fast Genetics, IA50010, Ames, USA; 3 Iowa Pork Industry Center, Iowa State University, Ames, IA 50010 USA

**Keywords:** Genetic parameters, Vulva size, Reproduction, Gilts, GWAS, Genomic prediction

## Abstract

**Background:**

Reproductive performance is critical for efficient swine production. Recent results indicated that vulva size (VS) may be predictive of reproductive performance in sows. Study objectives were to estimate genetic parameters, identify genomic regions associated, and estimate genomic prediction accuracies (GPA) for VS traits.

**Results:**

Heritability estimates of VS traits, vulva area (VA), height (VH), and width (VW) measurements, were moderately to highly heritable in Yorkshire, with 0.46 ± 0.10, 0.55 ± 0.10, 0.31 ± 0.09, respectively, whereas these estimates were low to moderate in Landrace, with 0.16 ± 0.09, 0.24 ± 0.11, and 0.08 ± 0.06, respectively. Genetic correlations within VS traits were very high for both breeds, with the lowest of 0.67 ± 0.29 for VH and VW for Landrace. Genome-wide association studies (GWAS) for Landrace, reveled genomic region associated with VS traits on *Sus scrofa* chromosome (SSC) 2 (154–157 Mb), 7 (107–110 Mb), 8 (4–6 Mb), and 10 (8–19 Mb). For Yorkshire, genomic regions on SSC 1 (87–91 and 282–287 Mb) and 5 (67 Mb) were identified. All regions explained at least 3.4% of the genetic variance. Accuracies of genomic prediction were moderate in Landrace, ranging from 0.30 (VH) to 0.61 (VA), and lower for Yorkshire, with 0.07 (VW) to 0.11 (VH). Between-breed and multi-breed genomic prediction accuracies were low.

**Conclusions:**

Our findings suggest that VS traits are heritable in Landrace and Yorkshire gilts. Genomic analyses show that major QTL control these traits, and they differ between breed. Genomic information can be used to increase genetic gains for these traits in gilts. Additional research must be done to validate the GWAS and genomic prediction results reported in our study.

## Background

Female reproductive traits, such as the number of piglets born, are well known to have low heritability, with estimates of around 0.10 [1]. Thus, genetic progress for improved performance for these traits is challenging. The identification of an indicator trait for reproductive performance in pigs could increase genetic gains for these traits. To be an indicator trait, it must be more heritable and have high genetic correlation with the trait of interest, in addition to being easy to measure. For instance, milk production in sows is difficult to measure directly, but can be indirectly estimated from piglet weight gain [2]. Finally, in the case of reproductive performance in pigs, an ideal indicator trait would be one that is phenotypically observable at a young age.

Recently, Romoser et al. [3] suggested that vulva size (VS), measured at 15 weeks of age in replacement gilts, could be used as an indicator trait for subsequent farrowing performance. These authors observed that gilts having large VS had lower culling rates (16% vs. 26%), greater first farrowing rates (78% vs. 60%), and greater number of piglets born alive at first parity (12 vs. 11.3) compared to gilts classified as small VS, indicating a clear and strong relationship between VS and reproductive performance [3]. Variation in VS in pre-pubertal gilts is associated with differences in ovarian follicular activity, suggesting that gilts with greater pre-pubertal ovarian activity will reach puberty at a younger age and have a greater VS at 15 weeks of age [4]. However, little is known about the genetic basis governing this association.

Knauer et al. [5] reported a high heritability for vulva width (VW) in gilts after reaching puberty, with an estimate of 0.57. These authors reported favorable, albeit weak, genetic correlation between VW and the probability of a gilt reaching first-parity (r_g_ = 0.07) and age at first farrowing (r_g_ = 0.24). In addition, these authors reported a negative genetic correlation between VW and total number of piglets born (r_g_ = − 0.33). Although it seems that there is clear genetic variation in VS traits in pigs, these correlations were weak and incongruent. Finally, the age of gilts when VS was measurement in Knauer et al. [5] were quite different than those proposed by Graves et al. [4] and Romoser et al. [3], indicating that the age may be an important factor to consider when measuring genetic variation for VS. Therefore, the objectives of this study were to (1) estimate genetic parameters for VS traits, (2) identify genomic regions associated with VS traits, and (3) estimate genomic prediction accuracies (GPA) for VS traits.

## Results

### Genetic parameters

Estimates of variance, common-environment effect (c^2^), and heritability are presented in Table 1 for each breed. For Landrace, heritability estimates were low to moderate, with 0.16 ± 0.09, 0.24 ± 0.11 and 0.08 ± 0.06 for VA, VH, and VW, respectively. For Yorkshire, these were moderate to high, with 0.46 ± 0.10, 0.55 ± 0.10 and 0.31 ± 0.09 for VA, VH, and VW, respectively. For all traits, there was a greater additive genetic variance in Yorkshire compared to Landrace gilts, although residual variances were similar between breeds. Estimates of c^2^ were low to moderate for Landrace, with 0.17 ± 0.05, 0.10 ± 0.05 and 0.22 ± 0.06 for VA, VH, and VW, respectively, and low for Yorkshire, with 0.05 ± 0.03, 0.04 ± 0.03 and 0.06 ± 0.03 for VA, VH, and VW, respectively,

**Table 1:** Estimates of residual (${\sigma}_e^2$), and additive genetic (${\sigma}_a^2$) variances^a^, common-environmental effect (*c*^2^), and heritability (*h*^2^) for vulva size traits by breed

Trait^b^	Landrace				Yorkshire			
	${\sigma}_e^2$	${\sigma}_a^2$	*c*^2^	*h*^2^	${\sigma}_e^2$	${\sigma}_a^2$	*c*^2^	*h*^2^
VA	89,166.2	21,953.3	0.17 (0.05)	0.16 (0.09)	71,990.1	69,807.7	0.06 (0.03)	0.46 (0.10)
VH	30.5	11.2	0.10 (0.05)	0.24 (0.11)	20.1	27.1	0.04 (0.03)	0.55 (0.10)
VW	20.8	2.5	0.22 (0.06)	0.08 (0.06)	22.6	10.8	0.06 (0.03)	0.31 (0.09)

^a^Expressed as *mm*^*4*^ for VA, and *mm*^*2*^ for VH and VW;

^b^*VA* vulva area, *VH* vulva height, *VW* vulva width

Estimates of genetic and phenotypic correlations between VS traits are presented in Table 2. All correlations were high and positive. Genetic correlations in Landrace gilts, with 0.99 ± 0.03 (VA and VH), 0.98 ± 0.04 (VA and VW), and 0.67 ± 0.29 (VH and VW), were overall numerically greater than in Yorkshire animals, which had 0.92 ± 0.03 (VA and VH), 0.93 ± 0.03 (VA and VW), and 0.73 ± 0.10 (VH and VW). Phenotypic correlations tended to be lower than genetic correlations in Landrace gilts, with 0.88 ± 0.01 (VA and VH), 0.90 ± 0.01 (VA and VW), and 0.61 ± 0.03 (VH and VW) whereas these were similar in Yorkshire gilts, which had 0.87 ± 0.01 (VA and VH), 0.90 ± 0.01 (VA and VW), and 0.61 ± 0.03 (VH and VW).

**Table 2:** Estimates (SE) of phenotypic (*r*_*P*_) and genetic (*r*_*G*_) correlations between vulva size traits

Trait 1	Trait 2	Landrace		Yorkshire	
		*r*_*P*_	*r*_*G*_	*r*_*P*_	*r*_*G*_
VA	VH	0.88 (0.01)	0.99 (0.03)	0.87 (0.01)	0.92 (0.03)
VA	VW	0.90 (0.01)	0.98 (0.04)	0.90 (0.01)	0.93 (0.03)
VH	VW	0.61 (0.03)	0.67 (0.29)	0.61 (0.03)	0.73 (0.10)

*VA* vulva area, *VH* vulva height, *VW* vulva width

### Genome-wide association analysis

**Fig. 1:**
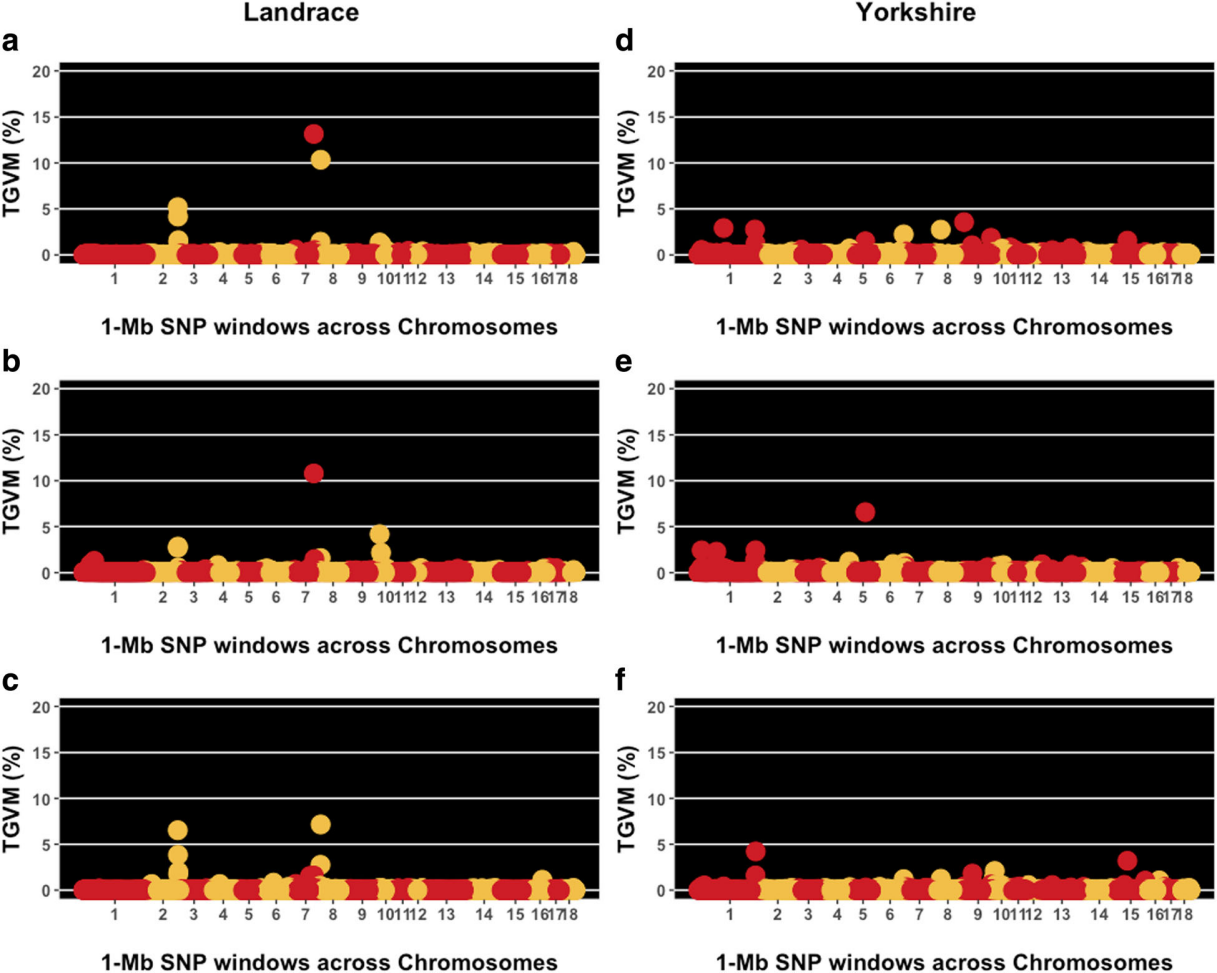
Manhattan plot for vulva size traits by breed. Each data point represents a 1-Mb SNP window plotted against the percentage of total genetic variance accounted for by the markers (TGVM, %). The chromosomes (1 to 18) and 1-Mb SNP window locations are ordered from left to right. Plots **a**, **b**, and **c** represent results for vulva area (VA), height (VH), and width (VW), respectively, for Landrace breed. Plots **d**, **e** and **f** represent results for VA, VH, and VW, respectively, for Yorkshire breed

Results from GWAS for both breeds are presented in Fig. 1 and Table 3. For each breed, we identified common QTL regions from overlapping window intervals and close proximity across VS traits. For Landrace, the *Sus scrofa* chromosome (SSC) 2 (154–157 Mb) revealed a common QTL region among VA, VH, and VW which accounted for 12.9, 6.2, and 15.8% of the total genetic variance accounted for by the markers (TGVM), respectively. There was a common QTL region on SSC 7 (107–110 Mb) between VA and VH, which accounted for 14.0% (VA) and 13.5% (VH), whereas on SSC 10 (8–19 Mb) a common QTL region accounted for 4.7% (VA) and 8.7% (VH). A common QTL region on SSC 8 (4–6 Mb), was found between VA and VW, which accounted for 11.1 and 10.3% TGVM, respectively.

**Table 3:** Genomic regions associated with vulva size traits by breed

Breed	Trait^a^	SSC^b^	Mb^c^	No. of SNP^d^	%TGVM^e^	PPI^f^
Landrace	VA	2	154–157	119	12.9	0.90
		7	107–110	64	14.0	0.73
		8	4–6	112	11.1	0.76
		10	8–19	355	4.7	0.61
	VH	2	154–157	119	6.2	0.63
		7	107–110	64	13.5	0.70
		10	8–19	355	8.7	0.75
	VW	2	154–157	119	15.8	0.79
		8	4–6	112	10.3	0.64
Yorkshire	VA	1	282–287	154	4.7	0.58
	VH	1	87–91	64	4.8	0.54
		1	282–287	154	3.4	0.50
		5	67	34	6.8	0.70
	VW	1	282–287	154	6.9	0.68

^a^*VA* vulva area, *VH* vulva height, *VW* vulva width;

^b^*Sus scrofa* chromosome;

^c^Megabase location of the SNP window;

^d^Number of SNPs in the SNP window;

^e^Total genetic variance accounted for by the markers;

^f^Posterior probability of inclusion of the SNP window

For Yorkshire gilts, there were fewer QTL identified for VS traits. A common QTL region among VA, VH, and VW on SSC 1 (282–287 Mb) accounted for 4.7, 3.4, and 6.9% TGVM, respectively. For VH, there was a QTL located on SSC 1 (87–91 Mb) which accounted for 4.8% TGVM and one on SSC 5 (67 Mb), which accounted for 6.8% TGVM.

### Genomic prediction

**Fig. 2:**
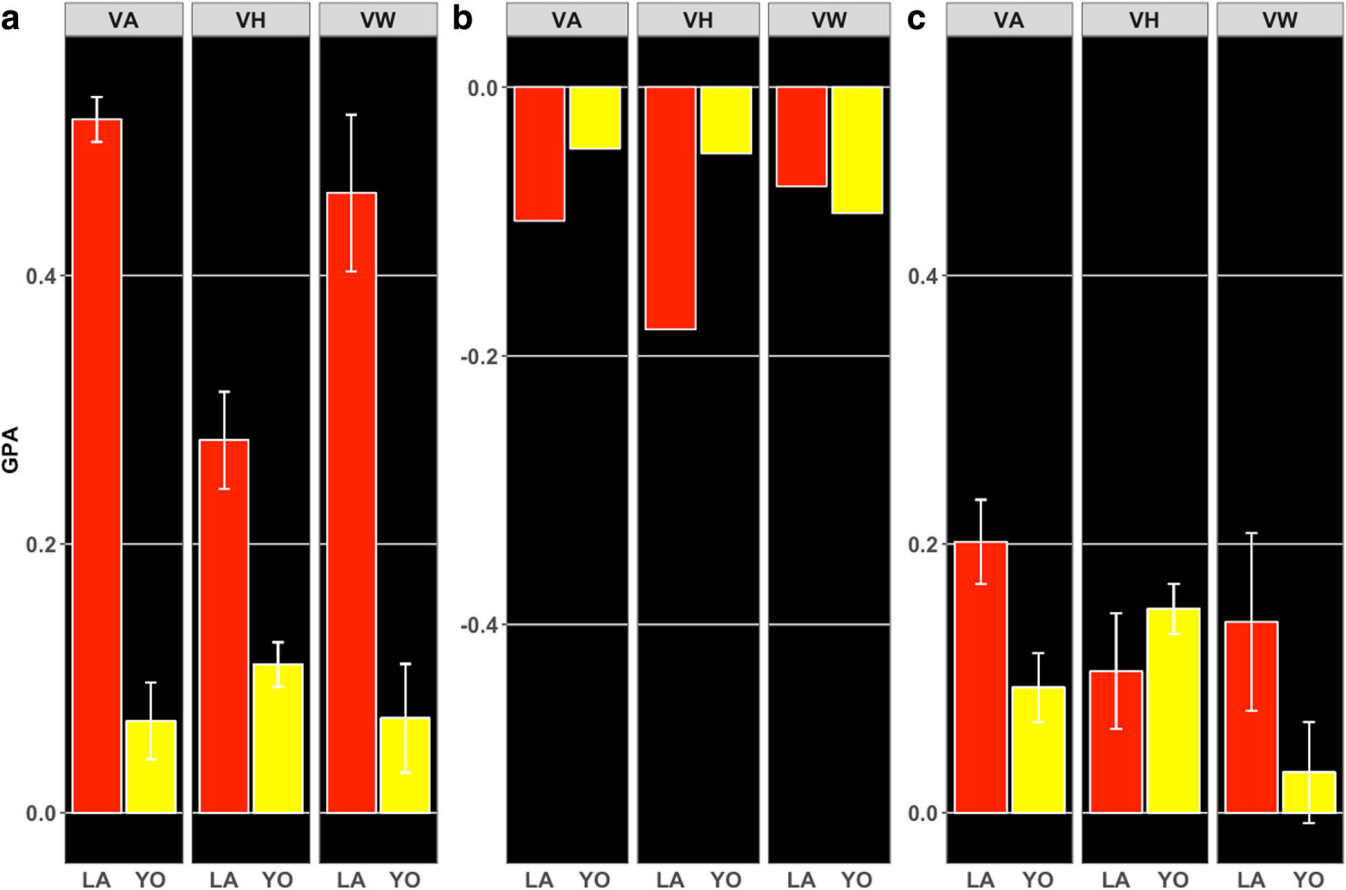
Genomic prediction accuracies (GPA) for vulva area (VA), vulva height (VH), and vulva width (VW). The x-axis represents the breed used as the validation group: Landrace (LA; red) and Yorkshire (YO, yellow). **a** Within-breed GPA, using 4- and 6-fold cross-validation for LA and YO, respectively, **b** Between-breed GPA, and **c** Multi-breed GPA, using 10-fold cross-validation, using one-fold per breed for validation at a time. Error bars represent the standard deviation of the GPA across folds

GPA are presented in Fig. 2. For within-breed analyses (Fig. 2a), GPAs (± SD) were moderate in Landrace, with 0.61 ± 0.02 (VA), 0.30 ± 0.04 (VH), and 0.52 ± 0.06 (VW), whereas these were lower in Yorkshire, with 0.07 ± 0.03 (VA), 0.11 ± 0.02 (VH), and 0.07 ± 0.04 (VW). In general, GPAs for between-breed (Fig. 2b) were low and consistently negative, with − 0.12 (VA), − 0.20 (VH), and − 0.08 (VW) when validating in Landrace and − 0.05 (VA), − 0.05 (VH), and − 0.10 (VW) when validating in Yorkshire. Multi-breed GPAs were overall low (Fig. 2c), with 0.24 ± 0.04 (VA), 0.12 ± 0.05 (VH), and 0.16 ± 0.07 (VW) when validating in Landrace gilts, and 0.10 ± 0.03 (VA), 0.16 ± 0.02 (VH), and 0.03 ± 0.04 (VW) when validating in Yorkshire gilts.

**Fig. 3:**
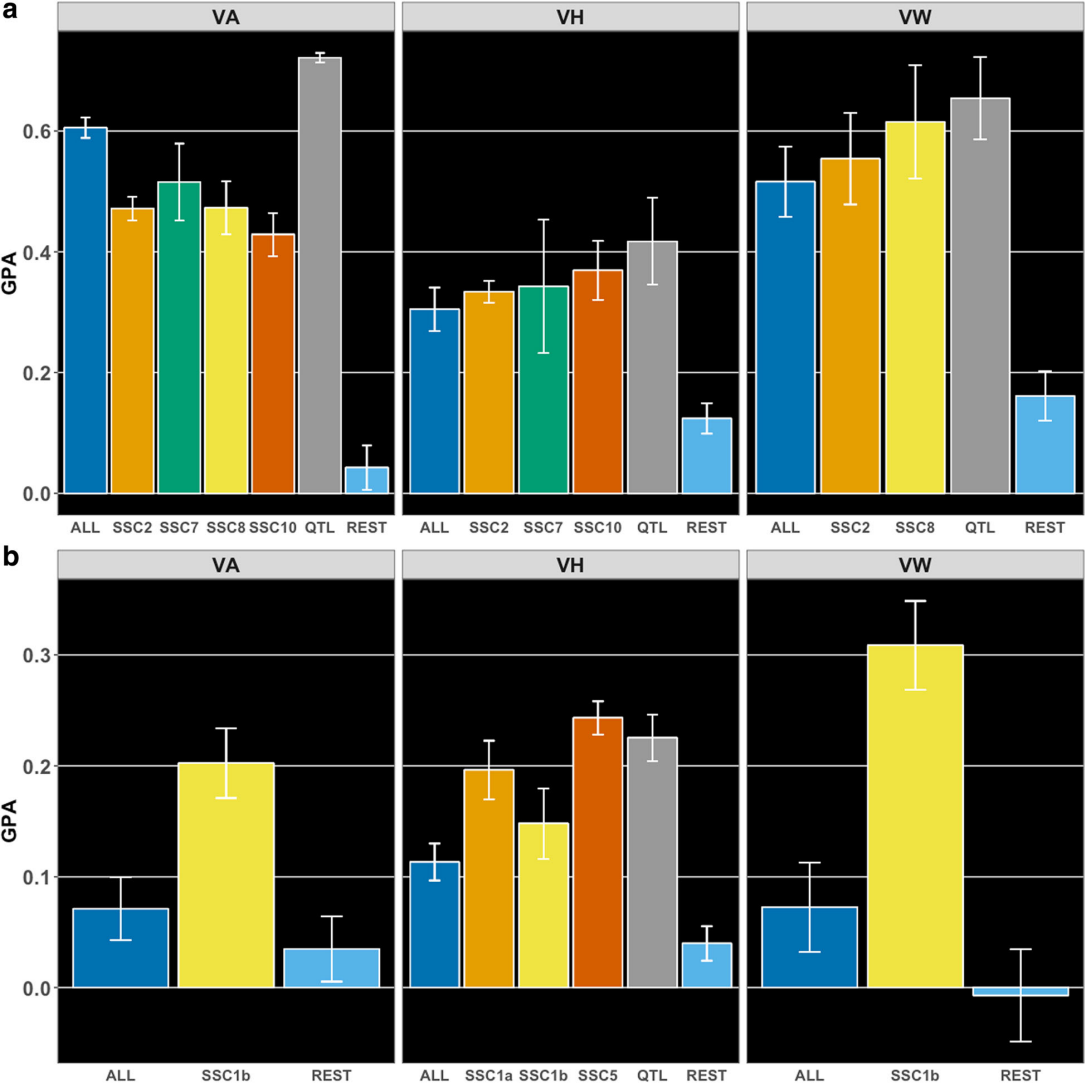
Genomic prediction accuracies (GPA) based on different sets of SNPs based on the GWAS results. Results for Landrace and Yorkshire breeds are in **a** and **b**, respectively, for vulva area (VA), vulva height (VH), and vulva width (VW). Within each panel, color bars represent GPA across SNP datasets. **a** For Landrace, *Sus scrofa* chromosome (SSC) 2, SSC 7, SSC 8, and SSC 10 represent the SNP datasets based on QTL identified on SSC 2 (154–157 Mb), 7 (107–110 Mb), 8 (4–6 Mb), and 10 (8–19 Mb), respectively. **b** For Yorkshire, SSC 1a, SSC 1b, and SSC 5 represent the SNP datasets based on QTLs on SSC 1 (87–91 Mb), SSC 1 (282–287 Mb), and SSC 5 (67 Mb), respectively. ALL represents all markers used for analysis (as presented in Fig. 1), QTL represents the SNP dataset based on all QTL identified for a given trait per breed, and REST represents the SNP dataset based on ALL minus the SNPs outside the QTL and neighboring upstream and downstream 3-Mb regions. Error bars represent the standard deviation of accuracies across cross-validation folds

Additional GPAs were estimated within breed based on SNPs within identified QTL (Fig. 3). For Landrace (Fig. 3a), moderate GPAs were obtained using SNPs from QTL identified on SSC 2, SSC 7, SSC 8, and SSC 10. Using QTL markers on SSC 2 (154–157 Mb), GPAs were 0.47 ± 0.02 (VA), 0.33 ± 0.02 (VH), and 0.55 ± 0.08 (VW). For SSC 7 (107–110 Mb), GPAs were 0.52 ± 0.06 (VA), and 0.34 ± 0.11 (VH). For SSC 8 (4–6 Mb), we found GPAs of 0.47 ± 0.04 (VA), and 0.62 ± 0.09 (VW). For QTL on dataset SSC 10 (8–19 Mb), GPAs were 0.43 ± 0.04 (VA), and 0.37 ± 0.05 (VH). In addition, we also evaluated the GPA when all SNPs located in these QTL regions were combined. From this strategy, we obtained moderate GPAs, with 0.72 ± 0.01, 0.41 ± 0.07, and 0.65 ± 0.07 for VA, VH and VW, respectively. Finally, we also evaluated the GPA for when markers not included in these QTL regions were used, in addition to removing neighboring SNPs at 3-Mb upstream and downstream regions (REST). GPAs for the REST dataset were low, with 0.04 ± 0.04, 0.12 ± 0.03, and 0.16 ± 0.04 for VA, VH and VW, respectively. For Yorkshire (Fig. 3b), we obtained low GPAs when using only QTL SNPs on SSC 1 and SSC 5. For SSC 1 (87–91 Mb), GPA was 0.20 ± 0.03 for VH. For SSC 1 (282–287 Mb), GPAs were 0.20 ± 0.03 (VA), 0.15 ± 0.03 (VH), and 0.31 ± 0.04 (VW). For SSC 5 (67 Mb), we found 0.24 ± 0.01 (VH). When using all QTL SNPs for VH, GPA was 0.23 ± 0.02. When using only SNPs outside pre-defined QTL regions (REST) GPAs were low, with 0.03 ± 0.03, 0.04 ± 0.02, and − 0.01 ± 0.04 for VA, VH, and VW, respectively.

## Discussion

In this study, we investigated the genetic and genomic bases of VS traits in Landrace and Yorkshire gilts. This study was motivated by the findings from Graves et al. [4], in which a positive correlation between VS and the presence of large ovarian follicles, indicating closer proximity to puberty onset, was discovered. Further Romoser et al. [3] proposed the use of VS as a selection tool to improve reproductive performance in sows However, there are very few studies available in the literature regarding the genetic basis of VS, and none, to the best of our knowledge, regarding the genomic basis of VS in pigs. The genetic identification of SNPs/QTL and candidate genes associated with VS traits could further enhance our understanding of the genetic and physiological mechanisms that result in variation in these traits.

### Genetic parameters

In general, VS traits were moderately to highly heritable in Yorkshire, and low to moderate in Landrace. Although residual variances for each trait were somewhat similar between breeds, additive genetic variances were consistently numerically smaller in Landrace, which could explain the lower heritability estimates in this breed. Allied to the high genetic correlations observed for both breeds, these results indicate that selection for changes in VS traits is possible in both breeds, and that changes in one VS trait would result in changes in other VS traits.

The heritability estimate for VW reported by Knauer et al. [5] was greater (0.57) than the ones obtained in the current study, with 0.08 and 0.31 for Landrace and Yorkshire, respectively. These authors measured VW of pubertal gilts with 162 days of age average. Although there are no other reports on genetic parameters for VS in pigs, to the best of our knowledge, there is relevant literature regarding the relationship between VS and reproductive performance. In Graves et al. [4], vulva measurements were utilized as developmental proxies for follicular activity. These authors observed prepubertal follicular development beginning between 75 and 115 days of age. Furthermore, a greater percentage of gilts with larger vulvas at 95 days of age reached puberty by 200 days of age compared to counterparts with smaller vulvas. Their results suggest that utilization of VS changes between 95 and 115 days of age could be a useful tool to identify replacement gilts prior to puberty. The age at which VS is measured might be an important consideration since the strength of the association between VS and puberty onset was lowered as gilts aged in Graves et al. [4], suggesting that there is a window of opportunity for which VS measurement is a reliable predictor of puberty onset. On the other hand, based on the work of See [6], selecting for age at puberty did not change the VW. However, VW measurements on the work of See [6] were taken during estrus, different than the work in Graves et al. [4], making these studies not directly comparable.

Although heritability estimates where larger in Yorkshire compared to Landrace, there was a greater c^2^ in Landrace compared to Yorkshire. Knauer et al. [5] also reported a low c^2^ for VW (0.05) in Landrace-LargeWhite gilts. Like in our Yorkshire data, the low c^2^ was accompained by a higher h^2^ in Knauer et al. [5]. This data set might be small to properly separate both components.

### Genome-wide association analysis

Genomic analysis identified genomic regions associated with VS traits (Fig. 1). In Landrace, we determined associated regions with VS on SSC 2, 7, 8, and 10. Of these, the same QTL on SSC 2 was found for VA, VH, and VW, the same QTL on SSC 8 was found for VA and VW, whereas the same QTL on SSC 7, and SSC 10 were observed for VA and VH. In Yorkshire gilts, we noted two regions associated with VS on SSC 1 and one on SSC 5. Of these, the same QTL on SSC 1 (282–287 Mb) was identified for VA, VH, and VW. Overall, the GWAS results had a consistent influence of SSC 2 and SSC 1 on VA, VH, and VW for Landrace and Yorkshire respectively. Thus, these results could suggest a pleiotropy mode of action for VS traits, which is in accordance with the high genetic correlation discovered in our study among these traits. Within these regions, we identified candidate genes and previously reported QTL for reproductive-related traits. These are reported in Table 4.

**Table 4:** Candidate genes and previously identified related QTL for genomic regions associated with vulva size traits

Breed	Trait^a^	SSC^b^	Mb^c^	Candidate genes	Related QTL
Landrace	VA, VH, VW	2	154–157	*PDGFRB*, *MGAT1*	Total number born, corpus luteum number [7], gestation length, mummified pigs [8].
	VA, VH	7	107–110	*FLRT2*, *SPATA7*, *TGFB3*, *IRF2BPL*	Teat number [9–12], uterine horn length [13], nonfunctional nipples [14].
	VA, VW	8	4–6	*MAN2B2*	Corpus luteum number [7, 15, 16], total number born alive, total number born [17], nonfunctional nipples [18], cryptorchidism [19], plasma FSH concentration [20], teat number [21].
	VA, VH	10	8–19	*ESRRG*, *TGFB2*	Number of stillborn [8], corpus luteum number [7], teat number [9].
Yorkshire	VH	1	87–91	*FYN*, *TSPYL1*, *TSPYL4*	Teat number [9, 22], total number born alive [8, 23], total number born, litter weight [23], mummified pigs [8].
	VA, VH, VW	1	282–287	*ACTL7A*, *ACTL7B*, *CTNNAL1*, *PTGR1*	Total number born, mummified pigs [8], teat number [9, 22, 24], age at puberty [10], left teat number, right teat number [22], corpus luteum number [7].
	VH	5	67	*CD9*, *GAPDH*, *AKAP3*	Teat number [17], uterine horn weight, reproductive tract weight, uterine horn length [25], corpus luteum number [7], litter weight [23].

^a^*VA* vulva area, *VH* vulva height, *VW* vulva width;

^b^*Sus scrofa* chromosome;

^c^Megabase location of the SNP window

For Landrace, within the QTL region on SSC 2 (154–157 Mb) for VA, VH, and VW, there are genes that could be related to reproductive development: was Platelet Derived Growth Factor Receptor Beta (*PDGFRB*), and Mannosyl (Alpha-1,3-)-Glycoprotein Beta-1,2-N (*MGAT1*). *PDGFRB* plays roles in regulation of embryonic development and angiogenesis [26–28]. Genetic variants of the *PDGFRB* gene have been associated with semen production traits in Chinese Holstein bulls [29]. Gene expression studies on *MGAT1* determined that this gene is involved in regulation of spermatogenesis and ovarian function in mice [30, 31], representing potential pathways affecting fertility. QTL for reproductive traits have been previously reported in this region, such as for total number born and corpus luteum number [7], and for gestation length and mummified pigs [8].

Several candidate genes are located within the SSC 7 QTL region (107–110 Mb) associated with VA and VH in Landrace: Fibronectin Leucine Rich Transmembrane protein 2 (*FLRT2*), Spermatogenesis Associated 7 (*SPATA7*), Transforming Growth Factor Beta 3 (*TGFB3*) and Interferon Regulatory Factor 2 Binding Protein Like (*IRF2BPL*). *FLRT2* has been identified to be required for embryonic development in using mice [32–36]. In a GWAS study using commercial Large White and Landrace pigs, *FLRT2* was proposed as a candidate gene for a QTL on SSC 7 (114.35–114.36 Mb) for number of piglets born alive [37], which further supports our hypothesis of VS traits being associated with farrowing performance. Multiples studies have reported *SPATA7* as having an important role in spermatogenesis in human, mouse, and rat [38–40]. This gene has also been suggested as a candidate gene for semen traits in a GWAS study using commercial Large White and Landrace [41]. Gene expression studies using mice and rats showed that *TGFB3* are involved in reproductive functions, such as gonadal and secondary sex organ development, spermatogenesis and ovarian function, immunoregulation of pregnancy, embryo implantation, and placental development [42–45]. In swine, the *TGFB* gene superfamily, which includes *TGFB3*, has been identified to be expressed in ovarian follicles of different sizes before and after in vitro culture in porcine oocytes collected from crossbred Landrace gilts at 155 days of age [46]. This study suggested that the *TGFB* gene superfamily is associated with the stage of maturation of porcine oocytes and the follicle size [46]. With regards to *IRF2BPL*, this gene encodes for a transcription factor that regulates neuronal networks controlling female reproductive function in nonhuman primates and rodents [47]. Inhibition of hypothalamic *IRF2BPL* delayed puberty, disrupted estrous cyclicity, and resulted in ovarian abnormalities [47]. On SSC 7, previous QTL reported in this region are teat number [9–12], uterine horn length [13], and nonfunctional nipples [14].

The Mannosidase Alpha Class 2B Member 2 (*MAN2B2*) gene is located within the QTL region (4–6 Mb) on SSC 8 associated with VA and VW in Landrace. This gene is involved in early spermatogenesis in pigs [48]. In addition, this gene has been proposed to be a candidate gene for ovulation rate in pigs, based on a QTL mapping study [15]. Additional relevant QTL have been previously identified in this region, such as for corpus luteum number [7, 15, 16], total number born alive, total number born [17], number of non-functional nipples [18], cryptorchidism [19], plasma FSH concentration [20], and teat number [21].

Within the QTL region associated with VA and VH on SSC 10 (8-19 Mb), are located the Estrogen Related Receptor Gamma (*ESRRG*) and Transforming Growth Factor Beta 2 (*TGFB2*) genes. *ESRRG* had been identified as a candidate gene involved in pubertal development on a GWAS study in beef cattle [49]. Monsivais et al. [50], in a review study of the *TGFB* family gene, described the influence of *TGFB2* over the reproductive function across several species. *TGFB2* gene is expressed during the peri-implantation and pregnancy periods in mice and humans. [50]. Jackowska et al. [46] studied the influence of *TGFB* family genes in swine and demonstrated that *TGFB2* is expressed in the porcine oocyte, suggesting that this family gene could be associated with maturation of porcine oocytes and follicle size. Additionally, relevant QTL have been previously reported in this region, for traits such as number of stillborn piglets [8], corpus luteum number [7], and teat number [9].

In Yorkshire gilts, two different regions on SSC 1 were associated with all VS traits evaluated in this study. Within 282–287 Mb are located the Actin Like 7A (*ACTL7A*), Actin Like 7B (*ACTL7B*), Catenin Alpha Like 1 (*CTNNAL1*), and Prostaglandin Reductase 1 (*PTGR1*) genes. *ACTL7A* and *ACTL7B* play functions related to capacitation of spermatozoa and fertility in mice [51, 52]. In Large White sows, it has been shown that *CTNNAL1* is associated with litter size in pigs [53, 54]. Association analysis of *CTNNAL1* with litter size in Large White pigs determined significant differences of total number born and number born alive among three genotypes, suggesting that *CTNNAL1* might be use as a reliable marker for pig selection and breeding [54]. Also, *PTGR1* is involved in maintenance of pregnancy in pigs [55]. Finally, QTL in this region have been previously reported for teat number [9, 22], total number born alive [8, 23], total number born, litter weight [23], and mummified pigs [8].

Within the other region on SSC 1 (87–91 Mb), which was identified for VH only, are located the FYN Proto-Oncogene (*FYN*), Testis Specific Protein Y-Linked 1 Like 1 (*TSPYL1*), and Testis Specific Protein Y-Linked 1 Like 4 (*TSPY4*) genes. *FYN* is involved in spermatogenesis in mice [56]. *TSPYL1* and *TSPYL4* are involved in male fertility in humans [57, 58]. QTL for reproductive traits have been previously reported in this region, such as for total number born, mummified pigs [8], teat number [9, 22, 24], age at puberty [10], left teat number, right teat number [22], and corpus luteum number [7].

Within the QTL region identified for VH on SSC 5 (67 Mb) are located the CD9 Molecule (*CD9*), Glyceraldehyde-3-Phosphate Dehydrogenase (*GAPDH*), and A-Kinase Anchoring Protein 3 (*AKAP3*) genes. The importance of *CD9* in fertilization of mammals has been previously discussed in relation to sperm-penetration, sperm-egg interaction and, and egg activation [59, 60]. An in vitro study with pig oocytes demonstrated that CD9 is expressed during early growth and meiotic maturation of oocytes, and participates in sperm-oocyte interactions during fertilization [59]. A gene expression analysis in the boar testis suggested that *GAPDH* is involved in spermatogenesis [61]. A study by immunolocalization techniques in bovine spermatozoa demonstrated that *AKAP3* plays an important role in modulating sperm functions [62]. Previous relevant QTL identified in this region include teat number [17], uterine horn weight, reproductive tract weight, uterine horn length [25], corpus luteum number [7], and litter weight [23].

In general, genomic regions identified in this study for VS traits include relevant genes for reproduction-related traits, as well as relevant QTL previously reported. Since there were no other genomic studies for VS available in the literature at the time of completion of this study, we were unable to validate the identified QTL. Interestingly, the regions identified in each breed were not identified in the other breed, indicating that the genomic architecture of these traits is quite unique to each of these populations. However, within each breed, the same QTL region was identified for multiple VS traits. The QTL regions on SSC 1 (282–287 Mb) and 2 (154–157 Mb) were found for the three traits investigated, for Yorkshire and Landrace, respectively. In addition, in Landrace, the QTL regions identified on SSC 7, 8, and 10 were found for more than one trait. Theses multiple hits within a region indicate a pleiotropic mode of action of these regions, which is supported by the high genetic correlation between these traits within each breed.

### Genomic prediction

**Fig. 4:**
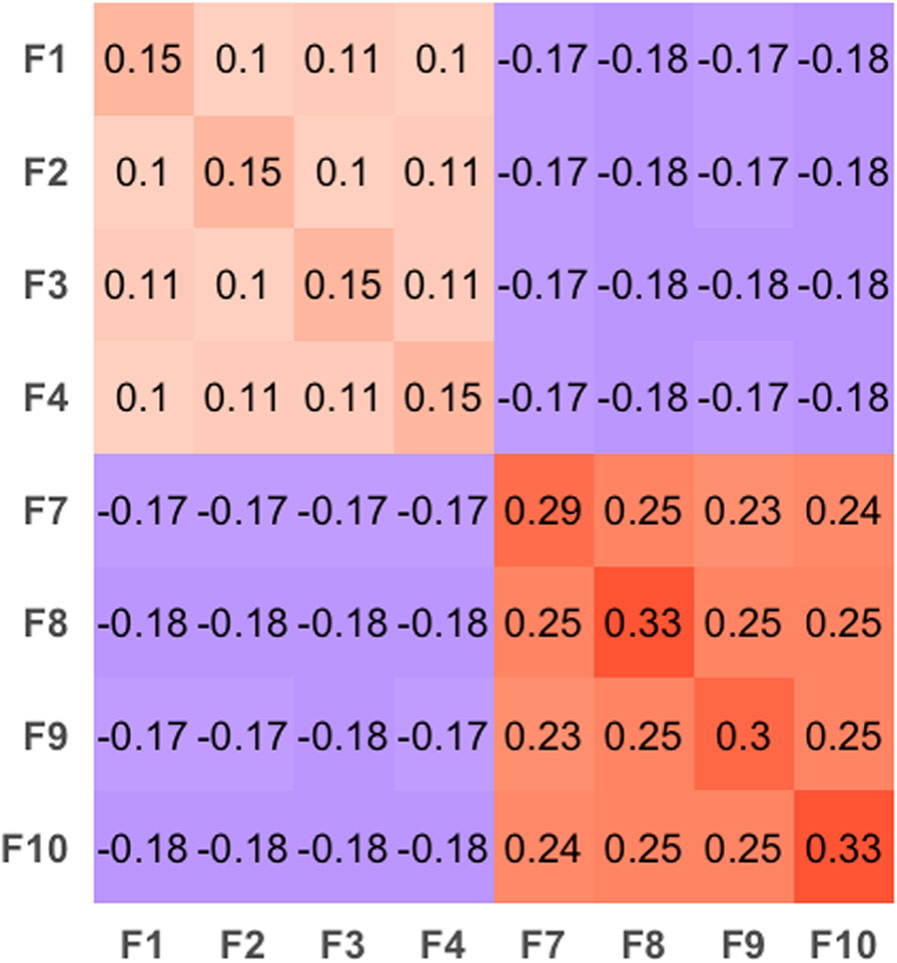
Heatmap of the average genomic relationships within (diagonal) and between (off-diagonals) cross-validation folds. Folds (F) F1 to F6 belong to Yorkshire, and F7 to F10 to Landrace breeds. Each number represents the average genomic relationships of individuals within (diagonal) and between (off-diagonals) folds, with boxes with positive and negative numbers in red and purple, respectively

In our study, genomic prediction results differed according to the training-validation strategy being used. For the within-breed genomic prediction analysis, we observed greater GPAs in Landrace compared to Yorkshire. In addition, we observed very low and negative results for the between-breed analysis. Therefore, we investigated the genomic relationship within and between cross-validation folds (Fig. 4). The greater GPAs in Landrace could be explained by the greater genomic relationships within and between the cross-validation folds for this breed, compared to the genomic relationships observed in Yorkshire. For example, the average within- and among-fold genomic relationships were 0.31 and 0.24 for Landrace, respectively, compared to 0.17 and 0.10 for Yorkshire, respectively. Therefore, it is expected a more accurate estimation of SNP effects in Landrace, because of the greater within-fold relationships, compared to those in Yorkshire pigs. Likewise, with the greater among-fold relationships in Landrace compared to Yorkshire, GPAs were expected to be then greater in Landrace than in Yorkshire. It has been shown that genomic predictions are more accurate if the genomic relationship between the validation and the training population is higher [63]. Another possible explanation is the fact that in Landrace, we found more QTL that are explaining a higher proportion of the genetic variance, suggesting that the genomic information is more capable of explaining the phenotype.

For between-breed genomic predictions, the low and negative GPA results are in accordance with the negative genomic relationships calculated between the folds of the two breeds. In addition, the GWAS results did not show any QTL associated within the same region between breeds. Therefore, with the negative and low relationships, and the lack of common QTL, between-breed analyses were expected to be low and negative. Nonetheless, results were consistently negative, which could indicate that small-effect QTL were captured while training SNPs, and that these might be in opposite phases between breeds.

For multi-breed genomic prediction, GPAs were lower than for the within-breed analysis for Landrace but not for Yorkshire. This indicates that increasing the dataset used for training only benefitted Yorkshire. However, this increased in GPA when validating in Yorkshire pigs was only marginal. In fact, there was a decrease in GPA from within- to multi-breed analyses for VW. These results are in accordance with the GWAS and between-breed results, which clearly show how the genomic bases of these traits differ between breeds. Therefore, adding another breed did not improve GPA, which is in accordance with previous results in the literature [64–66]. We should highlight that the proportion of Yorkshire pigs was greater than of Landrace in the training population. This should then bias the marker estimates towards those in the Yorkshire breed. In this sense it was expected a greater decrease in GPAs in Landrace compared to Yorkshire. However, GPAs from the multi-breed analyses were positive, as from the within-breed analyses, indicating that even with a greater proportion of Yorkshire pigs in the training dataset, results from validating on Landrace pigs were not as extremely impacted as for the between-breed analyses. Nonetheless, given the overall low and negative GPA for the between-breed analysis, the opposite signs for the markers causing this negative GPA should now be canceled out when both breeds are analyzed simultaneously.

The genomic prediction analyses using SNPs within the identified QTL showed overall greater GPA than for using all markers. This was true for all scenarios evaluated, with the exception for VA in Landrace, where GPA using only markers within each QTL did not result in greater GPA compared to ALL. However, once all of these markers were combined together, GPAs were always greater than using the whole genome, indicating that there is a benefit in only using QTL information when predicting VS traits. Likewise, once SNPs within QTL were not used during validation (i.e. REST), GPAs were very low, indicating that the major effects were included in the QTL SNPs. However, for VW in Landrace, REST had a GPA of 0.14 ± 0.04, which could suggest than additional small-effect QTL were being captured, albeit not identified in the previous analyses. In general, GPAs for each QTL SNP scenario were similar to each other within a trait and breed, always within 0.07 (between GPAs using markers on SSC 7 [107–110 Mb] and SSC 10 [8–19 Mb] for VA in Landrace). It is important to note that, in all analyses, all markers were used during training, while taking into consideration the marker selection process of BayesB. However, only those within their respective QTL were used for prediction. Therefore, all marker effect estimates were conditional on the whole genome. This is important to avoid bias in the marker estimates. These results are in accordance with other studies that estimated marker effects using all markers and then predicted breeding values based on QTL SNPs only [67, 68]. Therefore, these results indicate that genomic prediction for VS traits is possible in purebred pigs.

## Conclusion

In this study we characterized the genetic and genomic bases of VS traits at approximately 23 weeks of age in purebred gilts. Results suggest that VS traits are lowly to highly heritable in pigs, which may be different at different ages during gilt development. In general, VS traits were less heritable in Landrace compared to Yorkshire pigs. For both breeds, VS traits were highly genetically correlated, indicating that selection for one VS trait would result in major changes in the other VS traits. Several genomic regions associated with VS traits were identified. Common QTL were found for all VS traits, but these differ between breeds. This could indicate and corroborate the fact that these traits are all genetically similar. In addition, relevant candidate genes related to characteristics of development of the reproductive organs, reproduction and productive characteristics are located within the identified QTL in this study, supporting our findings. Results show that genomic selection for VS traits is possible in purebred pigs although results for Yorkshire show only limited predictive ability of using markers. In general, genomic prediction within breed is advised, and using only SNPs within QTL regions showed greater accuracies for all traits. Our findings suggest that genomic information can be used to increase genetic gains for these traits in gilts. Additional research must be done to validate the GWAS and genomic prediction results reported in our study, and also to evaluate the use VS traits as indicator traits for reproductive performance in pigs.

## Methods

Animal Care and Use Committee approval was not obtained for this study because analyses were performed on existing data obtained as part of routine data recording in a commercial breeding program. All farms in this study are operating in line with the regulations on protection of animals.

### Animals and phenotypes

A total of 475 Landrace and 708 Yorkshire gilts from two lines for each breed from a commercial farm (Fast Genetics, Saskatchewan, Canada) were used for this study. All animals were reared under the same controlled conditions. After the completion of this study, all animals remained in the herd for commercial production purposes. A 19-generation pedigree including 5749 individuals was available for these animals. The estimated mean inbreeding of animals in the pedigree, removing animals without any inbreeding, was of 2.8 and 4.5% for Landrace and Yorkshire, respectively.

At 23.8 (SD = 0.9) weeks of age, all animals had VS measurements collected using an Ultra Tech digital calipers (General Tools, Secaucus, NJ, USA), following the same measurement procedures described by Graves et al. [4] and Romoser et al. [3]. Measurements included VW and vulva height (VH). Vulva area (VA) was estimated as the product between VW and VH. These measurements were recorded by trained personnel within 5 consecutive weeks. All gilts had body weights (BW) measured on the same day that VS traits were recorded. Reproductive data were not available on animals used in this study. The summary statistics of these traits can be found by breed in Table 5.

**Table 5:** Summary statistics by breed

Statistics^a^	VA (*mm*^*2*^)	VH (*mm*)	VW (*mm*)	BW (*kg*)
*Landrace* (*n* = 475)				
Min	300	18	15	75.3
Max	2700	60	45	150
Mean	1014.7	36.2	27.3	119.6
SD	390.3	7.2	5.7	12.2
*Yorkshire* (*n* = 708)				
Min	247	19	13	79.5
Max	2842	60	53	160
Mean	984.5	35.9	26.8	121.4
SD	389.2	7.1	6.0	11.4

*VA* vulva area, *VH* vulva height, *VW* vulva width, *BW* body weight;

^a^*Min* minimum value, *Max* maximum value, *Mean* mean value, *SD* standard deviation

### Genotype data

DNA was isolated from tail or ear tissue using the ReliaPrep 96/KingFisher tissue kits (Promega, Madison, Wisconsin, USA). Individuals were genotyped using the PorcineSNP60 BeadChip. Prior to statistical analysis, genotyping quality was assessed, and samples/SNPs were removed. Genotypes with GenCall scores below 0.20 were replaced with the average genotype of the SNP within breed. Markers with minor allele frequencies below 1% were removed, and individual samples and SNPs with a call rate below 0.8 were excluded. The number of SNPs that remained in the data set was 37,155 SNPs and no animals were removed.

### Genetic parameters

Genetic parameters for VS traits were estimated for each breed separately using the following animal model:

$$ {Y}_{ijkl}=\mu +{L}_i+C{G}_j+B{W}_k+{a}_k+{d}_l+{e}_{ijkl} $$

where *Y*_*ijkl*_ is the observed phenotype of individual *k* at the *i*^th^ level of *L*_*i*_ and the *j*^th^ level of *CG*_*j*_; *L*_*i*_ is the *i*^th^ level of the fixed-effect of line; *CG*_*j*_ is the *j*^th^ level of the fixed-effect of contemporary group; *BW*_*k*_ is the linear covariate of body weight of the *k*^*th*^ animal; *a*_*k*_ is the animal random effect of the *k*^th^ animal, assuming ${a}_k\sim N\left(0,\boldsymbol{A}{\sigma}_a^2\right)$, where ***A*** is the additive numerator relationship matrix based on the pedigree; *d*_*l*_ is the random common-environment effect of the *l*^th^ litter, assuming ${d}_l\sim N\left(0,\boldsymbol{I}{\sigma}_d^2\right)$, where ***I*** is the identity matrix; and *e*_*ijkl*_ is the random error term associated with *Y*_*ijkl*_, assuming ${e}_{ijkl}\sim N\left(0,\boldsymbol{I}{\sigma}_e^2\right)$. Heritability and common-environment effect were estimated using a univariate model and correlations were estimated using a bivariate model. Genetic parameters were estimated using ASReml 4.0 [69]

### Genome-wide association analysis

In order to identify associations between genetic markers and VS traits, genome-wide association studies (GWAS) were performed by breed. In addition to fitting SNP effects as random effects in a multi-locus model, the model included the fixed effects of *L*, *CG*, and *BW* (covariate). Bayesian genomic prediction methods [70] were used to perform the GWAS analysis. For this, the estimates of additive genetic and residual variances obtained from the genetic parameter estimations were used as priors. BayesCπ was performed to estimate the proportion of SNPs with zero effect (π) on these data. The estimated π (0.99) value was then used in BayesC and BayesB. A total of 50,000 iterations were used in Gibbs sampling, with a burn-in of 5000 cycles. Analyses were performed in GenSel version 4.4 [71].

Putative candidate genes within identified QTL regions and in the neighboring upstream and downstream 3-Mb regions were identified based on the Sscrofa10.2 genome assembly, using the JBrowse tool from the National Animal Genome Research Program (https://www.animalgenome.org/jbrowse/). QTL regions explaining at least 4% TGVM were discussed in this study, including the identification of candidate genes within these QTL.

### Genomic prediction

Genomic predictions of VS traits were performed using BayesB, BayesC, and BayesC0 (π = 0) using the same model described for GWAS. GPA were estimated using three training and validation strategies: (1) within breed, (2) between-breed, and (3) multi-breed. For within and multi-breed strategies, an *n*-fold cross-validation was used, in which *n*-1 folds were used for estimating SNP effects (i.e. training) while the remaining fold was used as the validation dataset. This was repeated until all *n* folds were used for validation. For within breed, 4- and 6-fold cross-validation were used for Landrace and Yorkshire, respectively. For multi-breed, a total of 10 folds (the sum of the within-breed folds) were used for cross-validation. In this strategy, the validation dataset included data on only one breed, whereas SNP effects were trained using both breeds. These folds were created based on sire families. In each fold, daughters from 5 randomly selected sires were grouped in order to increase the relationship within folds. The average (SD) number of animals in each fold was 119 (8.7) and 118 (12.3) for Landrace and Yorkshire, respectively.

An additional strategy was used for the within-breed approach based on the GWAS results. For this, genomic prediction was performed using different SNP sets. First, GPAs were calculated using all SNPs were used (referred as to ALL) using BayesB. Then, based on the results using ALL, SNPs sets were created based on QTL regions identified for each trait and breed. Therefore, depending on the trait and breed, different number of SNP sets were used. With this, GPAs were calculated using only SNP estimates and genotypes from each of these QTL, separately. Finally, GPAs were calculated using SNP estimates and genotypes from markers outside these QTL and referred to as REST. For REST, SNPs within 3 Mb from the limits of the QTL were removed to avoid SNPs in some degree of LD with the QTL to capture any unwanted effects [67].

GPA was calculated differently depending on the strategy. For the between-breed analysis, this was calculated as:

$$ GPA=\frac{r_{\left( GEBV,{y}^{\ast}\right)}}{\sqrt{h^2}} $$

Where ${r}_{\left( GEBV,{y}^{\ast}\right)}$ is the correlation between the genomic estimated breeding values (GEBV) and phenotypes adjusted for estimates of fixed-effects (*y*^∗^); and *h*^2^ is the heritability of the trait in the breed used for validation.

For the within- and multi-breed strategies, GPA was calculated as the weighted average across folds as:

$$ GPA=\frac{\frac{\sum_{i=1}^{folds}{n}_i{r}_{i\left( GEBV,y\ast \right)}}{\sum_{i=1}^{folds}{n}_i}}{\sqrt{h^2}} $$

Where *r*_*i*(*GEBV*, *y*)_ is the correlation of GEBV with *y*^∗^ of the *i*^th^ fold, *n*_*i*_ is the number of animals in the *i*^th^ fold, and *h*^2^ is the trait heritability estimate of the breed used in the validation dataset.

## Data Availability

The data that support the findings of this study are available from Fast genetics, but restrictions apply to the availability of these data, which were used under license for the current study, and so are not publicly available. Data are however available from the authors upon reasonable request and with permission of Fast genetics.

## Abbreviations

*ACTL7A*: Actin Like 7A
*ACTL7B*: Actin Like 7B
*AKAP3*: A-Kinase Anchoring Protein 3
ALL: All markers used in an analysis
BW: Body weight
*CD9*: CD9 Molecule
CG: Contemporary group
*CTNNAL1*: Catenin Alpha Like 1
DNA: Deoxyribonucleic acid
*ESRRG*: Estrogen Related Receptor Gamma
*FLRT2*: Fibronectin Leucine Rich Transmembrane protein 2
*FYN*: FYN Proto-Oncogene
*GAPDH*: Glyceraldehyde-3-Phosphate Dehydrogenase
GEBV: Genomic estimated breeding values
GPA: Genomic prediction accuracies
GWAS: Genome-wide association studies
*IRF2BPL*: Interferon Regulatory Factor 2 Binding Protein Like
L: Breed line
*MAN2B2*: Mannosidase Alpha Class 2B Member 2
Mb: Mega base
*MGAT1*: Mannosyl (Alpha-1,3-)-Glycoprotein Beta-1,2-N
*PDGFRB*: Platelet Derived Growth Factor Receptor Beta
*PTGR1*: Prostaglandin Reductase 1
QTL: Quantitative trait loci
REST: SNPs at 3-Mb upstream and downstream of a certain genomic region.
rg: Genetic correlation
SD: Standard deviation
SNP: Single nucleotide polymorphism
*SPATA7*: Spermatogenesis Associated 7
SSC: *Sus scrofa* chromosome
*TGFB2*: Transforming Growth Factor Beta 2
*TGFB3*: Transforming Growth Factor Beta 3
TGVM: Total genetic variance accounted for by the markers
*TSPYL1*: Testis Specific Protein Y-Linked 1 Like 1
*TSPYL4*: Testis Specific Protein Y-Linked 1 Like 4
VA: Vulva area
VH: Vulva height
VS: Vulva size
VW: Vulva width
